# IMPACT OF SARCOPENIA ON GAIT INDEPENDENCE IN OLDER ORTHOPAEDIC PATIENTS: A COMPARISON OF 2 DIAGNOSTIC ALGORITHMS

**DOI:** 10.2340/jrm.v57.42051

**Published:** 2025-03-28

**Authors:** Taiki IKEMOTO, Mitsunori TOKUDA, Yuki MORIKAWA, Kotoha KURODA, Naoki NAKAYAMA, Naho TERADA, Misuzu NIINA, Daisuke MATSUMOTO

**Affiliations:** 1 Department of Rehabilitation Heisei Memorial Hospital, Kashihara City, Nara Prefecture, Japan; 2 Graduate School of Health Sciences, Kio University, Kitakatsuragi-gun, Nara Prefecture, Japan

**Keywords:** sarcopenia, muscle skeletal, gait, inpatients

## Abstract

**Objective:**

This study aimed to compare the impact of sarcopenia on gait recovery using the Sarcopenia Special Interest Group of the International Society of Physical and Rehabilitation Medicine (ISarcoPRM) and the Asian Working Group for Sarcopenia 2019 (AWGS2019) algorithms in older orthopaedic patients.

**Design:**

A prospective observational study.

**Patients:**

A total of 153 orthopaedic patients (78.4% women; average age 79.3 ± 6.7 years) were included during hospitalization.

**Methods:**

Sarcopenia was defined using the ISarcoPRM and AWGS2019 algorithms on admission. Functional ambulation categories assessed gait independence before admission and on discharge. The impact of sarcopenia on worsened gait independence on discharge was evaluated using multivariate logistic regression analysis.

**Results:**

Sarcopenia based on the ISarcoPRM algorithm (prevalence=56.2%) was significantly associated with worsened gait independence (odds ratio: 3.94, 95% confidence interval: 1.51–10.25, *p* = 0.005), unlike sarcopenia based on AWGS2019 (prevalence=36.6%).

**Conclusion:**

Sarcopenia assessed using the ISarcoPRM algorithm was associated with worsened gait independence on discharge in older orthopaedic patients.

## LAY ABSTRACT

In the Asian Working Group for Sarcopenia 2019 (AWGS2019) sarcopenia diagnostic algorithm, skeletal muscle mass is assessed using bioelectrical impedance analysis. In contrast, the Sarcopenia Special Interest Group of the International Society of Physical and Rehabilitation Medicine (ISarcoPRM) sarcopenia diagnostic algorithm uses ultrasound images to assess skeletal muscle mass. In this study we compared the impact of 2 sarcopenia diagnostic algorithms, using different methods to assess skeletal muscle mass, on gait recovery in older orthopaedic patients during hospitalization. The results showed that sarcopenia assessed using the AWGS2019 algorithm was not associated with worsened gait independence on discharge; however, there was a significant association between worsened gait independence and sarcopenia when assessed using the ISarcoPRM algorithm. Thus, sarcopenia assessed using the ISarcoPRM algorithm could be a prognostic indicator of gait independence on discharge in older orthopaedic patients during hospitalisation.

Sarcopenia negatively affects the postoperative mortality rate and functional recovery in orthopaedic patients (1). During acute hospitalization, accurately assessing the appendicular skeletal muscle mass index (ASMI) using bioelectrical impedance analysis (BIA) according to the Asian Working Group for Sarcopenia 2019 (AWGS2019) algorithm is often impractical owing to metal implants, oedema, or postural difficulties (2, 3). Moreover, gait independence is associated with handgrip strength, but not with muscle mass measured by BIA (4). Ultrasound can assess muscle mass without interference from implants or oedema (5), and anterior thigh muscle thickness may be a more sensitive indicator of sarcopenia than the ASMI (6). Considering its importance for mobility, the Sarcopenia Special Interest Group of the International Society of Physical and Rehabilitation Medicine (ISarcoPRM) algorithm, proposed in 2020, includes anterior thigh muscle thickness instead of the ASMI (7). However, the association between sarcopenia using the ISarcoPRM algorithm and gait independence is unclear. Therefore, this study aimed to compare the impact of sarcopenia using the ISarcoPRM and AWGS2019 algorithms on gait independence on discharge in older orthopaedic patients during hospitalization.

## METHODS

This prospective observational study included 812 orthopaedic patients who were admitted between April 2022 and December 2023 and discharged by March 2024. The inclusion criteria were age ≥65 years and pre-admission Functional Ambulation Category (FAC) score ≥3 (ambulator, dependent on supervision) (8). Exclusion criteria were patients with neurological complications and missing data on muscle mass and gait independence.

The ASMI was assessed by BIA using a body composition analyser (mBCA 525; Seca, Hamburg, Germany) in the supine position (9), with low muscle mass defined as per the AWGS2019 as <7.0 kg/m² for men and <5.7 kg/m² for women (10). Anterior thigh muscle thickness was assessed using ultrasound images (LOGIQ e V2; GE Healthcare, Chicago, IL, USA) and a linear probe (12 L-RS; GE Healthcare) in B mode at 8 MHz. The limb was placed in a supine position at the midpoint between the anterior superior iliac spine and the proximal patella, with the skin sufficiently exposed. The thicknesses of the rectus femoris and vastus intermedius were measured from the short-axis images of the anterior thighs, and the sonographic thigh adjustment ratio (STAR) was calculated as the sum of the 2 divided by the body mass index (BMI) (6). According to the ISarcoPRM algorithm (7), low muscle mass was defined as <1.4 mm/BMI in men and <1.0 mm/BMI in women.

Handgrip strength was measured using a Smedley digital handgrip strength tester (GRIP-D; Takei Ltd, Tokyo, Japan) with maximum effort in the sitting position and elbow extension position. Both sides were measured twice, and the maximum value was used. In the AWGS2019 algorithm (10), low muscle strength using handgrip strength is defined as <28 kg in men and <18 kg in women, and in the ISarcoPRM algorithm (7), <32 kg in men and <19 kg in women. Physical function measures, such as gait speed and the chair–stand test, were classified as having low physical function because all patients had difficulty in gait independence or needed a walking aid during admission. Sarcopenia was defined as meeting both low muscle mass and muscle strength criteria in each algorithm.

Gait independence was assessed using the FAC (8) on an ordinal scale before admission and on discharge: ambulator, dependent on physical assistance (2 score or level); ambulator, dependent on supervision (3 points); ambulator, independent, level surface only (4 points); and ambulator, independent (5 points). The patients whose FAC was maintained or improved on discharge compared with that before admission were classified as the improved-gait group; those whose FAC had worsened were classified as the worsened-gait group. BMI was categorized into 3 groups: <18.5 kg/m² (underweight), 18.5–24.9 kg/m² (normal weight), and ≥25 kg/m² (obesity). Cognitive function and nutritional status were also assessed as covariates.

All assessments on admission were performed within 3 days of admission for those who did not undergo surgery and within 3 days postoperatively for those who underwent surgery. Statistical analysis was performed to compare the improved- and worsened-gait groups using Student’s *t*-test for parametric data, Mann–Whitney *U* test for non-parametric data, and χ^2^ and Fisher’s exact tests for nominal data. Sensitivity analysis was performed to compare the surgical and non-surgical groups. The impact of each sarcopenia algorithm on worsened gait independence on discharge was compared using multivariate logistic regression analysis adjusted for age, sex, surgery, BMI category, malnutrition, and cognitive decline. All analyses were performed using JASP for Windows (version 0.18.1; University of Amsterdam, Amsterdam, the Netherlands). Statistical significance was set at 0.05.

## RESULTS

A total of 153 (women: 78.4%, average age: 79.3±6.7 years, and average length of stay 51.0±25.9 days) participants were included in the analysis. The diseases included 98 fractures (52 involving hips; 32, vertebrae; 8, pelvic bones; and 6, other lower extremities), 38 arthroplasties (knees and hips), and 17 lumbar spinal stenoses and disc herniations. A total of 110 patients (71.9%) underwent surgery. The prevalence of sarcopenia was 56.2% and 36.6% according to the ISarcoPRM and AWGS2019 algorithms, respectively. The proportion of patients with worsened gait independence on discharge was 26.8% (Table I).

**Table I:** Patient characteristics and comparisons between improved- and worsened-gait independence groups during hospitalization at baseline

Factor	Total (*n*=153)	Improved (*n*=112)	Worsened (*n*=41)	*p*-value
Age	80.0 (74.0–84.0)	**78.0 (72.0–83.0)**	**83.0 (81.0–87.0)**	<0.001*
65–74 years	45 (29.4)	**41 (36.6)**	**4 (9.8)**	0.003***
75–84 years	74 (48.4)	51 (45.5)	23 (56.1)	
≥85 years	34 (22.2)	20 (17.9)	14 (34.1)	
Sex, women	120 (78.4)	86 (76.8)	34 (82.9)	0.42***
Length of stay (days)	47 (31–66)	**40 (28–59)**	**60 (44–89)**	<0.001*
Handgrip strength (kg)	16.3 (12.4–21.7)	**17.8 (13.6–23.0)**	**12.9 (9.3–16.1)**	<0.001*
Low muscle strength (ISarcoPRM)^a^	119 (77.8)	**80 (71.4)**	**39 (95.1)**	0.002****
Low muscle strength (AWGS2019)^b^	112 (73.2)	**75 (67.0)**	**37 (90.2)**	0.004***
STAR (mm/BMI)	0.95 ± 0.26	**0.99 ± 0.26**	**0.83 ± 0.20**	<0.001**
Low muscle mass (ISarcoPRM)^c^	108 (70.6)	**73 (65.2)**	**35 (85.4)**	0.02***
ASMI (kg/m^2^)	5.90 (5.41–6.72)	5.97 (5.44–6.82)	5.75 (5.26–6.58)	0.37*
Low muscle mass (AWGS2019)^d^	70 (45.8)	49 (43.8)	21 (51.2)	0.41***
Sarcopeniae				
ISarcoPRM	86 (56.2)	**53 (47.3)**	**33 (80.5)**	<0.001***
AWGS2019	56 (36.6)	37 (33.0)	19 (46.3)	0.13***
FAC score on hospital admission^f^	5 (5–5)	5 (5–5)	5 (4–5)	0.31*
3	8 (5.2)	**8 (7.1)**	**0 (0)**	0.02***
4	29 (19.0)	16 (14.3)	13 (31.7)	
5	116 (75.8)	88 (78.6)	28 (68.3)	
FAC score on hospital discharge^f^	5 (4–5)	**5 (5–5)**	**4 (3–4)**	<0.001*
2	2 (1.3)	**0 (0)**	**2 (4.9)**	<0.001***
3	25 (16.4)	8 (7.1)	17 (41.5)	
4	36 (23.5)	14 (12.5)	22 (53.6)	
5	90 (58.8)	90 (80.4)	0 (0)	
FAC score worse	41 (26.8)			
Disease				
Fracture (hip, vertebra, pelvic, and others)	98 (64.1)	**65 (58.0)**	**33 (80.4)**	0.03***
Arthroplasty (hip and knee)	38 (24.8)	34 (30.4)	4 (9.8)	
Lumbar spinal stenosis and disc herniation	17 (11.1)	13 (11.6)	4 (9.8)	
Surgery	110 (71.9)	84 (75.0)	26 (63.4)	0.16***
MMSE score	27 (25–29)	**28 (25.8–30)**	**25 (24–29)**	0.002*
Cognitive decline^g^	13 (8.5)	**6 (5.4)**	**7 (17.1)**	0.05****
BMI (kg/m^2^)	22.9 ± 4.0	23.2 ± 4.1	22.1 ± 4.0	0.13**
<18.5 (Underweight)	24 (15.7)	16 (14.3)	8 (19.5)	0.25***
18.5–24.9 (Normal weight)	88 (57.5)	62 (55.4)	26 (63.4)	
≥25.0 (Overweight/obese)	41 (26.8)	34 (30.4)	7 (17.1)	
MNA-SF score	9 (7–10)	**9 (8–10)**	**8 (6–10)**	0.005*
Malnutrition^h^	41 (26.8)	**24 (21.4)**	**17 (41.5)**	0.02***
ECW/TBW (%)	50.0 ± 3.6	**49.4 ± 3.52**	**51.6 ± 3.3**	0.001**

Data are presented as median (25^th^–75^th^ percentile), *n* (%), or mean ± standard deviation.

ASMI: appendicular skeletal muscle index; STAR: sonographic thigh adjustment ratio; ISarcoPRM: the Sarcopenia Special Interest Group of the International Society of Physical and Rehabilitation Medicine; AWGS: Asian Working Group for Sarcopenia; FAC: Functional Ambulation Categories; MMSE: Mini-Mental State Examination; BMI: body mass index; MNA-SF: Mini Nutritional Assessment Short Form; ECW/TBW: extracellular water/total body water.

* Mann–Whitney *U* test,

** Student’s *t*-test,

*** χ^2^ test,

**** Fisher’s exact test; bold values indicate *p*<0.05.

a Low muscle strength: males <32 kg, females <19 kg (ISarcoPRM cut-off value).

b Low muscle strength: males <28 kg, females <18 kg (AWGS2019 cut-off value).

c Low muscle mass: STAR males <1.4 mm/BMI, females <1.0 mm/BMI (ISarcoPRM cut-off value).

d Low muscle mass: ASMI males <7.0 kg/m^2^, females <5.7 kg/m^2^ (AWGS2019 cut-off value).

e Sarcopenia: low muscle mass+low muscle strength (ISarcoPRM or AWGS2019 cut-off value).

f FAC categories: 2: ambulator, dependent on physical assistance; 3: ambulator, dependent on supervision; 4: ambulator, independent, level surface only; and 5: ambulator, independent.

g Cognitive decline: MMSE score ≤21.

h Malnutrition: MNA-SF score ≤7.

Compared with the patients in the improved-gait group, those in the worsened-gait group had significantly higher age, extracellular water/total body water (ECW/TBW), and sarcopenia prevalence based on the ISarcoPRM algorithm (*p*<0.001) and a significantly lower FAC on discharge (*p*<0.001) (Table I).

Compared with the patients who did not undergo surgery, those who underwent surgery had a significantly higher FAC on admission (*p*=0.02) and discharge (*p*<0.01), higher ECW/TBW (*p*=0.03), and younger age (*p*=0.02) (Table SI).

Multivariate logistic regression analysis showed that in the ISarcoPRM model, only sarcopenia was significantly associated with worsened gait independence on discharge (odds ratio [OR]: 3.94, 95% confidence interval [CI]: 1.51–10.25, *p*=0.005). In the AWGS2019 model, with the age group of 65–74 years as reference, the age groups of 75–84 years (OR: 4.43, 95% CI: 1.35–14.59, *p*=0.02) and ≥85 years (OR: 5.37, 95% CI: 1.48–19.54, *p*=0.02) were significantly associated with worsened gait independence on discharge; however, sarcopenia as defined by the AWGS2019 algorithm was not significantly associated with worsened gait independence on discharge (OR: 1.08, 95% CI: 0.42–2.80, *p*=0.88) (Table II).

**Table II:** Multivariate logistic regression analyses for gait-independence outcomes in older orthopaedic patients

Item	OR	95% CI	*p*-value
ISarcoPRM model			
Age (75–84) (Ref: 65–74)	2.87	0.81–10.19	0.10
Age (≥85) (Ref: 65–74)	3.35	0.87–12.96	0.08
Women (Ref: Men)	1.52	0.52–4.44	0.44
Surgery (Ref: No surgery)	0.71	0.30–1.70	0.44
Underweight (Ref: Normal weight)	0.48	0.15–1.53	0.21
Obesity (Ref: Normal weight)	0.62	0.22–1.78	0.38
Malnutrition (Ref: No risk)	2.32	0.87–6.15	0.09
Cognitive decline (Ref: No risk)	2.15	0.59–7.93	0.25
Sarcopenia (ISarcoPRM) (Ref: Robust)	**3.94**	**1.51–10.25**	**0.005**
AWGS2019 model			
Age (75–84) (Ref: 65–74)	**4.43**	**1.35–14.59**	**0.02**
Age (≥85) (Ref: 65–74)	**5.37**	**1.48–19.54**	**0.02**
Women (Ref: Men)	1.09	0.38–3.10	0.88
Surgery (Ref: No surgery)	0.89	0.38–2.08	0.79
Underweight (Ref: Normal weight)	0.56	0.17–1.87	0.35
Obesity (Ref: Normal weight)	0.67	0.24–1.88	0.45
Malnutrition (Ref: No risk)	2.18	0.82–5.84	0.12
Cognitive decline (Ref: No risk)	2.68	0.74–9.68	0.13
Sarcopenia (AWGS2019) (Ref: Robust)	1.08	0.42–2.80	0.88

Multivariate logistic regression analyses, bold values indicate *p*<0.05.

Ref: reference group; OR: odds ratio; CI: confidence interval; ISarcoPRM: the Sarcopenia Special Interest Group of the International Society of Physical and Rehabilitation Medicine; AWGS: Asian Working Group for Sarcopenia.

## DISCUSSION

Sarcopenia assessed using the AWGS2019 algorithm was not associated with worsened gait independence on discharge; however, there was a significant association between worsened gait independence and sarcopenia assessed using the ISarcoPRM algorithm. A previous cross-sectional study (6) reported a significant correlation between the STAR and physical function. This is the first longitudinal study, to our knowledge, to depict that sarcopenia assessed by the ISarcoPRM algorithm using the STAR is more strongly associated with gait independence than that assessed by the AWGS2019 algorithm using the ASMI.

The prevalence of sarcopenia showed a difference of approximately 20% between the 2 algorithms. This could be because the quadriceps muscle thickness assessed by ultrasound could be more sensitive to age-related muscle mass loss than the ASMI by BIA (11, 12). In addition, no significant association was observed between sarcopenia based on the AWGS2019 algorithm and gait recovery. The ECW/TBW ratio was significantly higher in the worsened-gait group than that in the improved-gait group and was also significantly higher in patients who underwent surgery than that in those who did not. These results suggest that the ASMI may have been overestimated in the worsened-gait group owing to postoperative oedema (13, 14).

In conclusion, sarcopenia assessed using the ISarcoPRM algorithm was associated with worsened gait independence on discharge in older orthopaedic patients during hospitalization and could be used as a prognostic indicator of gait independence.

## Supplementary Material

IMPACT OF SARCOPENIA ON GAIT INDEPENDENCE IN OLDER ORTHOPAEDIC PATIENTS: A COMPARISON OF 2 DIAGNOSTIC ALGORITHMS
