# Supplementary material for: IMPACT OF SARCOPENIA ON GAIT INDEPENDENCE IN OLDER ORTHOPAEDIC PATIENTS: A COMPARISON OF 2 DIAGNOSTIC ALGORITHMS
Source: J Rehabil Med. 2025 Mar 28;57:42051. doi: 10.2340/jrm.v57.42051 (PMC11971940; doi:10.2340/jrm.v57.42051)
Supplement: IMPACT OF SARCOPENIA ON GAIT INDEPENDENCE IN OLDER ORTHOPAEDIC PATIENTS: A COMPARISON OF 2 DIAGNOSTIC ALGORITHMS [file JRM-57-42051-s1.pdf]

Supplementary material has been published as submitted. It has not been copyedited, or typeset by Journal of Rehabilitation Medicine

Table SI. Patient characteristics and comparison between non-surgical and surgical groups during hospitalisation at baseline.

Data are presented as median (25th–75th percentile), n (%), or mean ± standard deviation.

ASMI, appendicular skeletal muscle index; STAR, sonographic thigh adjustment ratio; ISarcoPRM, the Sarcopenia Special Interest Group of the International Society of Physical and Rehabilitation Medicine; AWGS, Asian Working Group for Sarcopenia; FAC, functional ambulation categories; MMSE, Mini-Mental State Examination; BMI, body mass index; MNA-SF, Mini Nutritional Assessment Short Form; ECW/TBW, extracellular water/total body water.

	Non- Surgical (n = 43)	Surgical (n = 110)	P value
Age (years)	**81.0 (79.5–84.5)**	**79.0 (73.0–84.0)**	**0.02***
65－74	**6 (13.9)**	**39 (35.5)**	**0.04*****
75－84	**26 (60.5)**	**48 (43.6)**	
≥85	**11 (25.6)**	**23 (20.9)**	
Sex women	33 (76.7)	87 (79.1)	0.76***
Length of stay (days)	46 (25–62)	47 (32–66)	0.44*
Handgrip strength (kg)	15.4 (11.6–19.4)	16.7 (12.5–22.1)	0.50*
low muscle strength (ISarcoPRM) [a]	36 (83.7)	83 (75.5)	0.39****
low muscle strength (AWGS2019) [b]	34 (79.1)	78 (70.9)	0.31***
STAR (mm/BMI)	0.97 ± 0.27	0.94 ± 0.25	0.44**
low muscle mass (ISarcoPRM) [c]	28 (65.1)	80 (72.7)	0.35***
ASMI (kg/m^2)	5.65 (5.24–6.75)	6.01 (5.46–6.71)	0.13*
low muscle mass (AWGS2019) [d]	25 (58.1)	45 (40.9)	0.06***
Sarcopenia [e]			
ISarcoPRM	24 (55.8)	62 (56.4)	0.95***
AWGS2019	19 (44.2)	37 (33.6)	0.22***
FAC score at hospital admission [f]	**5 (4–5)**	**5 (5–5)**	**0.02***
3	**5 (11.6)**	**3 (2.7)**	**0.03*****
4	**11 (25.6)**	**18 (16.4)**	
5	**27 (62.8)**	**89 (80.9)**	

FAC score at hospital discharge	**4 (3–5)**	**5 (4–5)**	**0.002**[*]
2	**1 (2.3)**	**1 (0.9)**	**0.02**[***]
3	**13 (30.2)**	**12 (10.9)**	
4	**11 (25.6)**	**25 (22.7)**	
5	**18 (41.9)**	**72 (65.5)**	
FAC score worse	15 (34.9)	26 (23.6)	0.16[***]
Disease			
Fracture (hip, vertebra, pelvic and others)	**40 (93.0)**	**58 (52.7)**	**<0.001**[***]
Arthroplasty (Hip and Knee)	**0 (0)**	**38 (34.6)**	
Lumbar spinal stenosis and disc herniation	**3 (7.0)**	**14 (12.7)**	
MMSE score	26 (24－29)	28 (25－29)	0.15[*]
Cognitive decline [g]	6 (14.0)	7 (6.4)	0.20[****]
BMI (kg/m^2)	22.3 ± 3.7	23.1 ± 4.2	0.29[**]
<18.5	8 (18.6)	16 (14.5)	0.35[***]
18.5－24.9	27 (62.8)	61 (55.5)	
≥25.0	8 (18.6)	33 (30.0)	
MNA-SF score	8 (6.5–10)	9 (8–10)	0.11[*]
Malnutrition [h]	16 (37.2)	25 (22.7)	0.07[***]
ECW/TBW (%)	**49.0 ± 3.7**	**50.4 ± 3.5**	**0.03**[**]

[*]: Mann-Whitney U test, [**]: Student's t-test, [***]: chi-square test, [****]: Fisher's exact test; bold values indicate $p < 0.05$.

[a] low muscle strength: male <32 kg, female <19 kg (ISarcoPRM cut-off value).

[b] low muscle strength: male <28 kg, female <18 kg (AWGS2019 cut-off value).

[c] low muscle mass: STAR male <1.4 mm/BMI, female <1.0 mm/BMI (ISarcoPRM cut-off value).

[d] low muscle mass: ASMI male <7.0 kg/m^2, female <5.7 kg/m^2 (AWGS2019 cut-off value).

[e] Sarcopenia: low muscle mass + low muscle strength (ISarcoPRM or AWGS2019 cut-off value).

[f] FAC categories: 2: ambulator, dependent on physical assistance; 3: ambulator, dependent on supervision; 4: ambulator, independent, level surface only; and 5: ambulator, independent.

[g] Cognitive decline: MMSE score ≤ 21.

[h] Malnutrition: MNA-SF score ≤ 7.
